# Case of a Cyst Containing Hydatids, Extracted from the Right Anterior Ventricle of the Brain of a Cow

**Published:** 1792

**Authors:** William Moorcroft

**Affiliations:** Veterinarian Surgeon in London.


### IV. *Case of a Cyst containing Hydatids, extracted from the right anterior Ventricle of the Brain of a Cow. Communicated in a Letter to Dr.* Simmons *by Mr.* William Moorcroft, *Veterinarian Surgeon in London.*

*To Dr.* SIMMONS.

SIR,

PRESUMING on the favourable reception which every attempt to improve the long-neglected study of the diseases of domestic animals appears to experience at present from the Public in general, but more particularly from medical practitioners, I am induced to request the insertion of the following case in the next volume of Medical Facts and Observations. Though it cannot be considered as containing any material practical improvement, yet its communication may be useful, inasmuch as it may excite a degree of more general attention to an object which has hitherto been very partially understood.

As we have few cases of this nature upon record, and as this may, on that account, become an object of occasional practical reference, I have entered into details which, perhaps, may appear trifling or ſuperfluous to many medical readers, but which notwithſtanding may be of ſome importance to thoſe who are more particularly intereſted in the ſubject.

I remain, SIR,

Your very obedient, humble Servant,

W. MOORCROFT.

### CASE.

It has been long known to anatomiſts, but more particularly to ſhepherds and butchers, that collections of a tranſparent colourleſs fluid, in one or more ſemi-opaque, thin, membranous capſules, generally of a ſpheroide figure, and of different ſizes, are occaſionally to be met with on the ſurface or within the ſubſtance of the brain of ſheep, particularly of thoſe of a certain age; and experience has uniformly proved that ſuch a collection conſtantly produces the death of the animal in which it is found, if it be left entirely to itſelf: yet notwithſtanding the ſymptoms characteriſing the preſence of ſuch a collection have been aſcertained by thoſe converſant with theſe animals, and in conſequence attempts have been made to relieve the affected animals, both by medical practitioners and others, others, it is yet to be regretted that the Public of this country have not been benefited by the communication either of the diagnoſtic ſymptoms, or of the reſults of the different experiments. It is true ſome old authors, in treatiſes on rural œconomy, have mentioned this circumſtance; but this has been done in ſo looſe a manner, that little, if any, dependence is to be placed upon the obſervations they have tranſmitted us.

Sheep are not the only domeſtic animals which are incident to theſe collections, though they appear to be more particularly ſo than any other claſs: cows are next moſt frequently ſubject to them, and they have been met with in dogs; but I am not acquainted, either from experience or record, with the occurrence of a ſimilar circumſtance in horſes, though the preſence of ſmall globular bodies in the plexus choroides of theſe animals is far from being unfrequent; but theſe have always appeared to me to bear a greater reſemblance to the *ſimple* * than

* By *ſimple hydatid* is meant an accumulation of water in a capſule compoſed of conſolidated laminæ of cellular membrane. By *animal hydatid* is underſtood a veſicular worm, or organiſed body, which enjoys life diſtinctly from that of the than to the *animal hydatid*, the ſubject of this paper.

The circumſtance of an animal having ſuch a collection within the cavity of the cranium, frequently turning in a circular direction, appears to have been conſidered as the characteriſtic, unequivocal ſymptom, and accordingly we find the diſeaſe, produced by the collection, called, in ſome parts of this country, the *gid*, in others, *turn*; in France, *tournoiement*, or *vertige*; in Italy, *ſtorno*, or *male vertiginoſo*. But as I am not yet in poſſeſſion of a ſufficient number of facts to enable me to give a ſatisfactory account of this diſeaſe, I ſhall confine myſelf to the relation of the caſe of a cow, in which I lately met with it.

Being at Ormſkirk, in Lancaſhire, in the beginning of September, 1791, I was deſired to ſee a cow, whoſe diſeaſe had baffled the endeavours of every one who had attempted to relieve her. She was a two-year-old heifer, of the long-horned or Lancaſhire breed, and had been the animal in which it is evolved, and which contains, or is contained in, a certain quantity of fluid.

There are many ſpecies of this worm, which have been named in alluſion to their mode of life, form, &c., as *ſolitary*, *ſocial*, *tæniaform*, *piſiform*, *aciniform*, &c.

always, until attacked by the prefent complaint, in apparently good health, and tolerable condition. On the 1ft of May, 1791, fhe was put to grafs in a fcore at fome diftance from the refidence of the proprietor, which prevented his feeing her for fix weeks, when he found her much worfe in condition, and continually rambling about the field without appearing difpofed to eat. That fhe might be under his immediate care, he had her removed to a little clofe near his own houfe, and made ufe of the different means pointed out by thofe he confulted. Here fhe remained till I faw her, without having been apparently benefited in the leaft degree by any thing which had been done for her.

I found the motions of her limbs in walking unufually flow, languid, and unfteady; her belly tucked up, her flank hollow, and, in fhort, her whole appearance announcing a ftate of confiderable emaciation and debility; yet, notwithftanding, fhe appeared to have a defire for food: her pulfe was rather weak, but regular; her refpiration natural, and the common evacuations were fuch as occur in a healthy ftate, except that the quantity was rather diminifhed.

It was obferved that fhe always carried her head very near the ground and was continually engaged either in walking near the hedges, or turning in a circular direction. On attending more clofely to this circumftance, I remarked, that when driven towards the right hand, fhe conftantly formed a circle of about three yards in diameter; and that, on the contrary, when tempted to go to the left, fhe kept at a little diftance from, and followed the line of, the hedges and ditches, and occafionally thruft her head, and particularly the right fide of it, againft projecting boughs and tall tufts of grafs.

She had been fo long accuftomed to ramble in a flow, but almoft continual, walk, in the way juft mentioned, that the field exhibited fome unufual appearances; in the middle the grafs was trodden perfectly flat, in nearly equally fized circular paths of about eighteen inches in breadth, and of the diameter before mentioned, whilft, on the contrary, on the fides there was only one path at about a yard from the fence, which followed exactly its different directions, and obferved generally the fame breadth and diftance.

The head of the animal was conftantly held obliquely downwards, fo that the left horn was confiderably more elevated than the right. On examining the head in general, I was not fenfible of the fmalleft morbid alteration either in feel feel or figure, but remarked that the right nostril appeared to yield a larger quantity of mucus, and the lining membrane to be somewhat redder than that of the left. The eyes were heavy, from the eye-lids being more closed than is usual; but I was not aware at this time of any diseased appearance in either of the pupils. The intervals from rambling, which were but of short duration, were employed in eating rather greedily.

On taking these different circumstances into consideration, I was induced to believe that the presence of one or more of the larvæ of the œstrus * in one or more of the nasal cavities might be the cause of these symptoms, and in consequence of this idea removed a circular piece of bone

* A fly well known to insectologists, and to be met with almost every where in the country in the autumnal months, but particularly in the neighbourhood of woods. It comes under the class *Diptera* of Linnæus:

" Alæ duæ. Halteres clavati, solitarii pone singulam " alam sub squamula propria.

" *Œstrus.* Os nullum, punctis tribus, absque Proboscide " aut Rostro exserto."—Vide Car. Linnæi Entomol. cur. Car. de Villers. 8vo. Lugdun. 1789. Tom. III. p. 345.

It should seem that this fly is a true parasite, and stands in need of an animal nidus during the states of egg and larva. Some species *(œstrus bovis)* deposit their eggs in the skin

 of bone from the upper part of the nasal, and another from the most depending part of the right maxillary sinus. The perfectly sound state of the membrane of these cavities convinced me of my error, and after being fully satisfied that the inflammation with which I was struck was only confined to a very small, and that the lower, portion, and probably produced by some external cause, I brought the flaps of skin into contact, and quitted the animal. This operation did not produce the smallest change in the symptoms; union of the divided tegument took place, and I lost sight of the animal till the latter end of the month, when the proprietor informed me that other assistance had been had recourse to, but without advantage, and that, unless I advised the trial of some farther means, he would have her killed, that I might have an of the backs of horned cattle and rein deer; others (*œstrus nasalis*) in the nasal cavities of ruminating animals, but particularly of sheep, and occasionally in the interstices of the folds of the lateral and upper part of the pharynx of the common or fallow deer; others again (improperly called *œstrus hæmorrhoidalis*) lay their eggs upon the hairs of the sides of horses, some of which are occasionally swallowed, and produce the larvæ commonly called botts, and which obtain in greater or less number in the stomach of almost every horse.

opportunity opportunity of being ſatisfied by diſſection as to the cauſe of the complaint. Unwilling to have this done without reviewing the ſymptoms, I re-examined the head with the greateſt accuracy, and found the pupil of the right eye more dilated than that of the left, though the latter was larger than it ought to have been; and though the former was not deſtitute of irritability, yet the latter enjoyed a greater latitude of motion.

From the addition of this to the former ſymptoms, I began to imagine that there might be an hydatid either upon the ſurface or within the ſubſtance of the brain, and was inclined to believe, that if ſuch was the caſe, there was a greater probability of detecting it by perforating that part of the ſkull correſponding with the right hemiſphere, than elſewhere.

The owner of the cow having conſented to whatever experiment or operation I might propoſe, I had her caſt, and ſecured by means of fetters, and made a crucial inciſion through the ſkin upon the right frontal bone, on a level with the ſuperior part of the orbit, and very near the longitudinal ſuture. After detaching the pericranium, I applied a trephine of nearly an inch diameter, and having removed two circular pieces of bone, I cut off the intercircular angles,

ſo

ſo as to reduce the whole to a longitudinal oval opening of ſomewhat more than two inches in length and one in breadth. Not meeting with any thing extraordinary upon the ſurface of the dura mater, I placed the ſcalpel upon it, in order to make an opening in it parallel with that of the bone, and was not a little ſurpriſed to find it oſſified; I, however, cut out a piece of it correſponding with the opening in the bone.

The veſſels of the pia mater appeared almoſt obliterated, or nearly colourleſs, from their containing an unuſually ſmall quantity of blood, and the expoſed ſurface of the brain preſented two conſiderable eminences ſeparated by a furrow, in which was a colourleſs vein which acted as a band, but yet not ſufficiently ſo as to prevent theſe eminences preſſing rudely againſt the edges of the bone.

On preſſing my finger on the brain, I received the ſenſation of fluid reſiſtance from within, and began a longitudinal inciſion in the upper and middle part of the denuded portion. The cortical part cut as if much upon the ſtretch, and was thinner and harder than uſual; on dividing the vein juſt mentioned, the lower part began to tear before the knife, and, when the whole inciſion was effected, the preſſure from within within became fo confiderable as to render the farther ufe of the cutting edge of the knife entirely unneceffary. By cautioufly feparating the divided edges with the handle of the inftrument, I was ftruck with the appearance of a cyft, part of which protruded itfelf immediately with confiderable force, through the bony opening, to the fize of a hen's egg, when it burft, and gave iffue to about three or four ounces of a thin colourlefs fluid. By laying hold of the torn edges, and drawing them gently from one fide to the other, I detached the cyft from its connections without the leaft degree of laceration, except what was before produced by the efcape of the contents.

On looking into the brain, after the removal of the cyft, I was only aware of a large cavity, the furface of which was perfectly fmooth and white, not containing any more hydatids, nor the fmalleft quantity of water. From the prefence of the plexus choroides, which lay at the bottom, though much paler coloured and fmaller than ufual, I concluded that this cavity was an enlargement of the right anterior ventricle, effected in all probability by the gradual extenfion of the contained cyft. I covered the opening with a piece of muflin, dried the flaps of the fkin well, well, replaced and covered them with a piece of linen, and fecured the whole with an adhefive plafter.

On removing the fetters the animal rofe without difficulty, and walked to her fhed without appearing in the leaft difpofed to turn or ramble.

That evening, about eight hours after the operation, fhe ate a fmall quantity of hay, and the next morning did not exhibit the fmalleft fymptom of derangement. Having fome affairs which called me to the continent, I was neceffitated to confide the animal to other care, and on my return, five months afterwards, was told that fhe died fixteen days after the performance of the operation. The account given me was, that fhe was very well for the firft five days, when fhe was dreffed; that on her appearing dull after the dreffing, the proprietor conceiving the bandage might be too tight, took it off, and replaced it; that in two hours fhe appeared to be re-eftablifhed, and ate and ruminated as ufual, but was not dreffed with any regularity afterwards; that on the twelfth day fhe became heavy, refufed her food, lay down, and fhewed fymptoms of pain and inquietude; and that on the fixteenth the proprietor, in compaffion for her fufferings, ordered her to be killed by opening the veſſels of the neck; it muſt, however, be obſerved, that this was done from a perſuaſion that her ſituation did not admit of recovery.

The opening of the head was effected by a blow of an axe, which produced ſuch a confuſion of parts as prevented a ſurgeon who was preſent from diſtinguiſhing any thing more than a very conſiderable quantity of maggots (*pupæ* of the fleſh fly) within the ſubſtance of the brain.

It is to be regretted that a proper attention was not given to this caſe; for, circumſtanced as it was, we cannot draw any concluſion from the practice which was adopted. Perhaps the ſuppuration of the brain would have deſtroyed the animal; but this ſuppuration was certainly increaſed by the admiſſion of air and by other cauſes of irritation.

The capſule or bag was thin, rather opaque, and tolerably ſtrong, without any appearance of vaſcularity; its external ſurface was in general ſmooth; in a few points, however, it was rendered irregular by the adheſion of ſmall, white globular bodies. The internal ſurface was in ſome places perfectly ſmooth, whilſt in others, on the contrary, it was ſtudded with groups of the bodies juſt mentioned, ſome of which were not larger than grains of poppy ſeed and nearly globular; globular; others, however, were as large as a ſmall pin's head, ſomewhat pyriform, and hung from the cyſt by a kind of neck. In ſome places they were ſcattered at a diſtance from each other, whilſt in others they were accumulated in ſuch numbers as to form cluſters, which hung down into the cavity of the capſule, and bore no ſlight reſemblance to ſmall bunches of grapes. Each of theſe bodies conſiſted of a veſicular worm, or animal hydatid, contained in a ſmall capſule, and which, from the circumſtance of its being found in great numbers in one common capſule, has been called the *ſocial hydatid*, to diſtinguiſh it from another ſpecies, which is generally met with iſolated, and thence named the *hermit* or *ſolitary hydatid*. This hydatid conſiſts of a head, neck, and body, and appears to be of the ſame ſtructure with the larger or ſolitary kind; but as I ſhall have occaſion to ſpeak of theſe worms in another paper, I ſhall reſerve what I have to ſay of their ſtructure and mode of life till that time.

Although I may riſk being cenſured for prolixity, yet I cannot avoid ſtating ſome of the cauſes which concur to render this complaint almoſt always fatal, and invariably highly dangerous.

These are,

1. The injury done to the brain by the presence of such a foreign body.

2. Its difference in situation and size.

The injury done to the brain may be considered as relative to the size and situation of the capsule.

Its different situation produces a degree of uncertainty and difficulty in ascertaining the part of the cranium necessary to be perforated. Its difference in size is an object of no small importance, as the degree of suppuration which must necessarily take place after the extraction of the capsule must be proportionate to the exposed surface of the brain.

The cyst is occasionally to be met with in every part both of the cerebrum and cerebellum. When seated superficially in the cerebrum, its pressure against the posterior surface of the part of the bone which is before it excites the action of the absorbents, which in a certain time remove the corresponding part of the bone, and a soft spot is left in its place, which serves as a guide to the operator *.

When

* There are occasional deviations from this course. In the case before us the efforts of nature appear to have been ill

When ſeated towards the baſis of the cerebrum, it meets with greater reſiſtance than when on the ſurface, increaſes in ſize, and produces a proportional diminution in the volume of the brain before it can effect the ſoftening of the correſponding part of the bone.

As I have not met with it in the cerebellum myſelf, I cannot ſay whether it is able to produce the ſoftening of the poſterior or any other part of the cranium, or whether the animal in which it is found is not deſtroyed before this effect takes place.

Its ſize may be conſidered as relative to its age and depth; for if it be deep ſeated, it muſt, by its extenſion, diſplace much brain before it can induce the ſoft ſpot; whilſt, on the contrary, if ſuperficial, the ſoft ſpot may take place without any very conſiderable extenſion of the capſule.

From what has been ſaid, it muſt appear that the moſt favourable caſes for the performance of the operation are thoſe in which the ſoft ſpot takes place ſoon after the appearance of the ill directed by ſtrengthening the dura mater inſtead of weakening it, and the bone had not undergone any perceptible change.

ſymptoms,

ſymptoms, in conſequence of the capſule being ſuperficial; but even here it muſt be undertaken with a very guarded prognoſtic.

The moſt unfavourable caſes are thoſe where either the ſoft ſpot does not make its appearance at all, or where it takes place after the other ſymptoms have been preſent for a conſiderable time, and where great debility and emaciation exiſt.

*Half-moon Street,*

*Piccadilly,*

March 30, 1792.

